# Functional Outcomes of a Dynamic Condylar Screw for Unstable Femoral Intertrochanteric Fractures in Patients Above 50

**DOI:** 10.7759/cureus.68905

**Published:** 2024-09-07

**Authors:** MohammedElhassan Abdalla, Hussein Samir Elrukby, Ahmed Mohamed, Monzir M Salih

**Affiliations:** 1 Department of Orthopaedics, Gezira Centre for Orthopedic Surgery and Traumatology, Wad Madani, SDN; 2 Department of Surgery, University of Gezira, Wad Madani, SDN; 3 Department of General Surgery, Burjeel Medical City, Abu Dhabi, ARE

**Keywords:** dynamic condylar screw, functional outcome, intertrochanteric, modified harris hip score, unstable fractures

## Abstract

Background: Hip fractures are among the most common fractures encountered in the emergency departments by orthopedic trauma teams. The optimal treatment method and implant choice for unstable intertrochanteric fractures are subject to debate, with various options available, including intramedullary and extramedullary implants.

Methods: In this descriptive cross-sectional study, the researchers examined patients with unstable intertrochanteric fractures (classified as 31A2 according to the AO Foundation/Orthopaedic Trauma Association (AO/OTA) classification) who had undergone open reduction and internal fixation with a 95° dynamic condylar screw (DCS). The study was conducted at the Gezira Centre for Orthopedic Surgery and Traumatology (GCOST) during the latter half of 2022. Functional outcomes were assessed using the modified Harris Hip Score (mHHS).

Results: A total of 30 patients were enrolled in this study, with a mean age of 73 ± 12.27 years. Of these, 11 (36.7%) were male, and 19 (63.3%) were female. The mHHS was 73.6 ± 14.654. Among the surveyed patients, seven (23.3%) reported poor outcomes, 13 (43.3%) reported fair outcomes, six (20%) reported good outcomes, and four (13.3%) reported excellent outcomes. The Kruskal-Wallis test revealed statistically differences in mean mHHS scores between gender groups (p = 0.024) and between age groups (p = 0.04). However, no significant differences were found across different modes of trauma groups (p = 0.73), affected hip groups (p = 0.35), comorbidity groups (p = 0.84), or postoperative complication groups (p = 0.06).

Conclusion: Our study found that DCS treatment for unstable intertrochanteric fractures yielded acceptable functional outcomes, making it a viable and effective treatment option.

## Introduction

Hip fractures are common injuries among older populations representing a severe and multifaceted burden. These fractures place a burden not only on the patients themselves but also on their families and the healthcare systems [1]. Additionally, elderly patients who sustain a hip fracture have a significantly higher risk of mortality compared to their age-matched counterparts. The risk is highest during the weeks and months following the fracture but remains elevated for years [2,3].

Hip fractures are disabling injuries that impair mobility, reduce autonomy, and increase the risk of recumbency-related health issues. Early surgical intervention to allow patient mobilization is essential to avoid these consequences. However, older patients frequently have comorbidities that need to be optimized before safely proceeding with the proposed surgery. Current treatment guidelines recommend surgical fixation of hip fractures within 24-48 hours of admission to the healthcare facility for better outcomes [4,5].

Intertrochanteric fractures comprise approximately 50% of all hip fractures. Typically, they are categorized into stable and unstable patterns. Unstable fractures, characterized by loss of the posteromedial buttress or lateral femoral cortex, present a significant challenge to surgeons in achieving stable fixation [6,7].

While the current guidelines by the American Academy of Orthopaedic Surgeons (AAOS) favor cephalomedullary nails over extramedullary devices for the treatment of unstable intertrochanteric fractures (AO Foundation/Orthopaedic Trauma Association (AO/OTA) type 31A2) [8], the National Institute for Health and Care Excellence (NICE) guidelines recommend the use of extramedullary devices in trochanteric fractures above and including the lesser trochanter except fractures with reverse oblique configuration [5].

However, even the latest Evidence-Based Clinical Practice Guideline from the AAOS Board of Directors recommends the conduction of high-level trials to compare cephalomedullary devices with sliding hip screws in a large group of patients with unstable intertrochanteric fractures. They are specifically encouraging trials to "assess pain, functional outcomes, radiographic outcomes, complications, and cost" [4].

Ultimately, there is an ongoing debate regarding the most effective methods for addressing unstable intertrochanteric fractures. On the ground, it is evident that the dynamic condylar screw (DCS) is considered a dependable option by many surgeons. Their trust in this device is demonstrated by the ongoing studies highlighting its usefulness, measuring its effectiveness, and comparing it to other surgical fixation methods used to treat unstable intertrochanteric fractures [9-12].

In Sudan, patients and their families bear the majority of the cost of operations since we lack comprehensive medical insurance to cover these expenses. As a result, patients often opt for DCS over the more expensive cephalomedullary devices. Additionally, the availability of the proximal femoral nail (PFN) is limited in many towns. Furthermore, as concluded by Dr. Daffalla Elshafie and Dr. Elwaleed Salim, esteemed senior orthopedic surgeons at the Gezira Centre for Orthopedic Surgery and Traumatology (GCOST): "The Dynamic Condylar Screw remains a dependable option for treating unstable intertrochanteric fractures, even though cephalomedullary nails are often recommended. It is important to emphasize that this option is not only accessible but also more affordable for the Sudanese population."

The cost and accessibility of healthcare services are two factors that consistently contribute to the loss of patients to follow-up [13]. In our context, these factors are expected to play a significant role in the remarkably high rates of patients lost to follow-up. To address this issue, and in the absence of institutional data, we consulted four local orthopedic surgeons. They helped estimate that most patients typically attend their six-month postoperative visit, which we established as the assessment point in our study.

This study aimed to investigate the functional outcomes of patients who received treatment with DCS for unstable intertrochanteric fractures at the GCOST. As the sole public hospital in the area offering specialized orthopedic and trauma care, the center treats a considerable number of patients from nearby rural areas and underprivileged states who have various musculoskeletal issues.

## Materials and methods

Study design and setting

This cross-sectional study was conducted at GCOST in Wad Madani, Sudan, during the second half of 2022. 

Ethical approval

Approval to conduct the study was obtained from GCOST, with approval number SUD24/GCOST-13. Additionally, informed consent was obtained from all patients included in the study.

Study population

The study included individuals aged 50 years and above who received initial surgical fixation using DCS for closed, unstable intertrochanteric femoral fractures (AO/OTA type 31A2) within three weeks of the causative event. These patients were followed for six months post-operation, with data collected at one point in time. Patients with pathological fractures or significant cognitive impairments were excluded. All the included patients had their surgeries supervised by one of the two senior orthopedic consultants with comparable expertise at the center.

Data collection and analysis

The total number of patients, fulfilling the inclusion criteria, was estimated from the records at the operation theatre department. The patients' demographic data along with relevant clinical information (independent variables) were documented and tabulated before the patients' scheduled visits. The modified Harris Hip Score (mHHS) questionnaire was filled out during the follow-up visits. Those who didn't show up at their appointment were called by telephone to complete the questionnaire. The data was collected at a single point in time, at six months postoperatively.

The independent variables of the study were the patients' age, gender, and comorbidities, mechanism of trauma, postoperative complications, and the side of the affected hip, while the dependent variable was the mHHS results.

The data was coded and analyzed using IBM SPSS Statistics for Windows, V. 25.0 (Released 2013, IBM Corp., Armonk, NY). Since our data did not follow a normal distribution, as determined by the Kolmogorov-Smirnov and Shapiro-Wilk tests, we used a nonparametric test for analysis (Kruskal-Wallis test).

## Results

A total of 67 patients underwent surgical fixation with a DCS for proximal femur fractures. Upon reviewing the operative notes, 13 (19.4%) patients were excluded from the study based on the following criteria: nine (13.4%) patients had subtrochanteric proximal fractures (AO/OTA type 31A3.2 and 32B2), one (1.5%) patient had a reverse oblique fracture pattern (AO/OTA type 31A3.1), and three (4.5%) patients were under 50 years old. Additionally, 14 (20.9%) patients were lost to follow-up, and 10 (14.9%) patients passed away before their six-month postoperative visit. The deaths were not caused by procedural complications or issues directly related to the device. Instead, they were attributed to other factors, such as the patient's overall health condition and comorbidities. Consequently, 30 (44.8%) patients were available and enrolled in the study. Among them, 27 (90%) patients attended the visit physically, while three (10%) patients filled out the questionnaire through telephone calls. 

The mean age of the participants was 73 years, with a female-to-male ratio of 1.7:1. The right hip was injured in 20 (66.7%) cases, and 26 (86.7%) fractures resulted from a simple fall from standing height. Additionally, 24 (80%) patients had no comorbidities, and 27 (90%) patients experienced no postoperative complications. Table 1 shows the study's independent variables. 

**Table 1:** Overview of the independent variables of the study The data are presented as mean ± SD, N, and % MVA: motor vehicle accident; HTN: hypertension; DM: diabetes mellitus; CVA: cerebrovascular accident; DVT: deep vein thrombosis

Independent variables		Characteristic	Count	Percent of participants
Age (in years)	Mean ± SD = 73 ± 12.27	50-64	7	23.3%
		65-74	8	26.7%
		75-89	10	33.3%
		90 and above	5	16.7%
Gender		Female	19	63.3%
		Male	11	36.7%
Mode of trauma		Simple fall from standing height	26	86.7%
		MVA	4	13.3%
Affected hip		Right hip	20	66.7%
		Left hip	10	33.3%
Comorbidities		No comorbidity	24	80%
		HTN and DM	3	10%
		DM	1	3.3%
		CVA	1	3.3%
		Bronchial asthma	1	3.3%
Postoperative complications		No complication	27	90%
		Superficial infection	1	3.3%
		Prominent implant	1	3.3%
		DVT	1	3.3%

The mean mHHS was 73.6 ± 14.654 (Table 2). Among the surveyed patients, seven (23.3%) reported poor, 13 (43.3%) reported fair, six (20%) reported good, and four (13.3%) reported excellent outcomes (Table 3).

**Table 2:** Overview of the mean mHHS mHHS: modified Harris Hip Score

Descriptive statistics					
	N	Minimum	Maximum	Mean	Std. deviation
Total mHHS	30	40	100	73.6	14.654

**Table 3:** Overview of the total mHHS mHHS: modified Harris Hip Score

mHHS	N (%)
Excellent	4 (13.3%)
Good	6 (200%)
Fair	13 (43.3%)
Poor	7 (23.3%)
Total	30 (100%)

For pain scoring, 19 (63.33%) patients reported no pain or disregarded their pain, and no patients reported serious limitations or being totally disabled. For gait assessment, 14 (46.66%) patients experienced only slight limping, while nine (30%) patients reported no limp. Additionally, 10 (33.33%) patients utilized a walking frame for support. In terms of walking distance, 12 (40%) patients could only walk indoors. In the evaluation of stair climbing, 11 (36.66%) patients indicated they were unable to climb stairs, whereas six (20%) patients were able to use various methods to navigate them, and another six (20%) patients could climb stairs normally. The majority of patients, 24 (80%), reported no difficulty wearing socks or shoes, and 21 (70%) patients could comfortably sit in any chair for an hour. Furthermore, 23 (76.66%) patients were able to use public transportation (Table 4).

**Table 4:** Overview of the pain assessment, gait function assessment, and functional activities assessment

Assessment		Item	N	%
Pain assessment	Pain	Totally disabled	0	0
		Marked, serious limitations	0	0
		Moderate, tolerable, makes concessions, occasional codeine	1	3.33
		Mild, no effect on ordinary activity, pain after activity, uses aspirin	2	6.66
		Slight, occasional, no compromise in activity	8	26.66
		None/ignores	19	63.33
Gait function assessment	Limp	Severe/unable to walk	4	13.33
		Moderate	3	10
		Slight	14	46.66
		None	9	30
	Support	Unable to walk	4	13.33
		2 crutches	0	0
		2 canes	0	0
		Crutch	10	33.33
		Cane, full time	7	23.33
		Cane, long walks	3	10
		None	6	20
	Distance walked	Bed and chair	4	13.33
		Indoors only	12	40
		2-3 blocks	7	23.33
		6 blocks	3	10
		Unlimited	4	13.33
Functional activities assessment	Stairs	Not able	11	36.66
		Any method	6	20
		Normally with banister	7	23.33
		Normally	6	20
	Stocks/shoes	Unable	2	6.66
		With difficulty	4	13.33
		With ease	24	80
	Sitting	Unable to sit, ½ hour, any chair	2	6.66
		High chair, ½ hour	7	23.33
		Any chair, 1 hour	21	70
	Transport	Unable to use public transportation	7	23.33
		Able to enter public transportation	23	76.66

The study revealed a significant decrease in functional outcomes with advancing age, as indicated by a p-value of 0.043 and a medium negative rank correlation coefficient (rho = -0.447), and also a statistically significant worse outcome was observed in females (p = 0.024; rho = -0.420). However, the functional outcome was not significantly affected by the mechanism of injury (p = 0.746) or whether the injury involved the right or left hip (p = 0.373). Similarly, the outcomes were not affected significantly by the comorbidities (p = 0.840) or postoperative complications (p = 0.065). Table 5 shows the correlation of mean mHHS across gender groups, mode of trauma groups, affected hip groups, comorbidity groups, complication groups, and age groups.

**Table 5:** Correlation of total mHHS to gender groups, mode of trauma groups, affected hip groups, comorbidity groups, postoperative complication groups, and age groups A p-value of less than 0.05 is considered significant mHHS: modified Harris Hip Score; min-max: minimum and maximum mHHS within the category; RTA: road traffic accident; CVA: cerebrovascular accident; DVT: deep vein thrombosis

Variables		mHHS (min-max)	Significance (p-value)
Gender	Males	82.0 (72.0-95.0)	0.024
	Females	72.0 (56.0-78.0)	
Mechanism of injury	RTA	76.0 (60.75-94.25)	0.73
	Simple fall	75.0 (69.50-82.25)	
Affected hip	Right hip	76.50 (71.0-82.75)	0.35
	Left hip	72.50 (63.0-80.0)	
Comorbidities	No comorbidities	76.50 (68.75-82.75)	0.84
	Diabetes	71.0 (71.0-71.0)	
	Diabetes and hypertension	70.0 (70.0-70.0)	
	CVA	78.0 (78.0-78.0)	
	Bronchial asthma	72.0 (72.0-72.0)	
Complication	No post-op complication	77.0 (71.0-77.0)	0.065
	Superficial infection	40.0 (40.0-40.0)	
	Prominent implant	65.0 (56.0-56.0)	
	DVT	44.0 (44.0-44.0)	
Age group	50-64 years	83.0 (75.0-92.0)	0.043
	65-74 years	77.0 (71.0-85.0)	
	75-89 years	71.0 (47.0-76.0)	
	90 years and above	70.0 (56.0-87.50)	
Overall mHHS		75.50 (69.50-82.25)	-

## Discussion

The management of unstable intertrochanteric fractures continues to be a challenge for orthopedic surgeons worldwide [14]. Several devices, including intramedullary nails, fixed-angle blade plates, dynamic hip screws (DHS), and DCS, are commonly used for these fractures [15]. Choosing the right device often depends on the fracture type, patient characteristics, and, in many cases, the availability of resources [9].

In this study, we used the mHHS, a well-validated tool, to assess postoperative hip function six months after surgery. The mean score was 73.6 ± 14.654, with nearly half of our patients reporting fair outcomes (43.3%). This aligns with findings from Ur Rehman et al. [16], who reported a similar mean mHHS of 65.4 ± 11.7 in a comparable patient population at six months postoperatively. Like Ur Rehman et al., we found that the DCS could be a reliable option for treating unstable proximal femoral fractures, providing satisfactory functional outcomes across a range of patients. Khan et al. [9] also supported this conclusion in their study of 84 patients, where outcomes were consistent with our findings.

Although the PFN may offer advantages such as less operative blood loss and shorter hospital stays [17], our use of the DCS was driven by practical factors. In Sudan, the selection of surgical devices is often dictated by cost and availability, and in many settings, the DCS remains an effective and accessible option. This consideration is critical, as the availability of more advanced fixation devices may not always be guaranteed in low-resource environments.

Interestingly, we observed significant differences in functional outcomes between male and female patients, with women tending to have worse scores. This highlights the potential influence of gender on postoperative recovery, possibly due to physiological differences such as muscle strength and bone density. By recognizing these differences, we can tailor rehabilitation programs to better address the unique needs of men and women.

On the other hand, we did not find significant differences in outcomes based on the mechanism of injury, the side of the hip affected, comorbidities, or postoperative complications. This suggests that patients, even those with additional health issues commonly seen in older populations, can still achieve reasonable functional outcomes following DCS treatment. This is an encouraging result for healthcare providers treating elderly patients with complex health conditions.

However, age did have a significant impact, with older patients showing worse functional outcomes. This reinforces the importance of age-specific care plans, which may involve more intensive rehabilitation and additional support for elderly patients to ensure the best possible recovery.

This study is the first to explore the use of DCS for unstable intertrochanteric fractures in Sudan. Given the limited data in this area, we hope our findings provide useful insights and encourage further research. Additionally, we suggest that future studies adopt an age range starting at 50 years to better capture the at-risk population for hip fractures, as our study showed that nearly a quarter of our patients fell within the 50-64 age.

The small sample size and short follow-up period may limit the generalizability of our findings. Future studies should include larger samples, longer follow-ups, and comparisons with alternative treatments. The study also did not document postoperative rehabilitation, which could affect functional outcomes.

## Conclusions

We conclude that managing unstable intertrochanteric fractures using a DCS is a practical and effective option, particularly in regions where a PFN is either unavailable or financially prohibitive. The DCS offers a reliable alternative for stabilizing such fractures, making it a valuable option in resource-limited settings where more advanced implants may not be accessible.
